# Noninvasive ventilation and exercise tolerance in heart failure: A systematic review and meta-analysis

**DOI:** 10.1590/bjpt-rbf.2014.0039

**Published:** 2014

**Authors:** Daiana C. Bündchen, Ana I. Gonzáles, Marcos De Noronha, Ana K. Brüggemann, Sabrina W. Sties, Tales De Carvalho

**Affiliations:** 1 Núcleo de Cardiologia e Medicina do Exercício, Centro de Ciências da Saúde e do Esporte, Universidade do Estado de Santa Catarina (UDESC), Florianópolis, SC, Brazil; 2 Universidade Federal de Santa Catarina (UFSC), Araranguá, SC, Brazil; 3 LA trobe University, Physical Therapy Bendigo, Victoria, Australia

**Keywords:** heart disease, exercise intolerance, positive pressure

## Abstract

**Background:**

Patients with heart failure (HF) usually develop exercise intolerance. In this context, noninvasive ventilation (NIV) can help to increase physical performance.

**Objective:**

To undertake a systematic review and meta-analysis of randomized controlled trials that evaluated the effects of NIV on exercise tolerance in patients with HF.

**Method:**

Search Strategy: Articles were searched in the following databases: Physiotherapy Evidence Database (PEDro), Scientific Electronic Library Online (SciELO), and MEDLINE. Selection Criteria: This review included only randomized controlled trials involving patients with HF undergoing NIV, with or without other therapies, that used exercise tolerance as an outcome, verified by the distance travelled in the six-minute walk test (6MWT), VO_2_peak in the cardiopulmonary test, time spent in testing, and dyspnea. Data Collection and Analysis: The methodological quality of the studies was rated according to the PEDro scale. Data were pooled in fixed-effect meta-analysis whenever possible.

**Results:**

Four studies were selected. A meta-analysis including 18 participants showed that the use of NIV prior to the 6MWT promoted increased distance, [mean difference 65.29 m (95% CI 38.80 to 91.78)].

**Conclusions:**

The use of NIV prior to the 6MWT in patients with HF may promote increased distance. However, the limited number of studies may have compromised a more definitive conclusion on the subject.

## Introduction

Heart failure (HF) is a complex clinical syndrome that results from impaired ventricular filling or ejection capacity^1^^,^ 2, and involvement of the left ventricle occurs in 60% of cases in adults^1^. 

Exertional dyspnea, termed exercise intolerance (EI), is the main symptom of HF and is closely linked to its pathophysiology^2^. Understanding the mechanisms of exercise intolerance is paramount in the treatment of HF because EI directly affects the diagnosis and reflects a poor prognosis for the patient^3^. Those mechanisms are multifactorial and remain poorly understood, and excessive ventilatory requirements -with predominantly restrictive ventilatory mechanics, inspiratory muscle fatigue, exacerbated muscle ergoreflex, accentuated sympathetic activity, or some combination of these factors - are prominent among them^3^. All these mechanisms contribute to the increase in central motor command to the respiratory muscles, withconsequent increases in perceived exertion and fatigue, similar to the results found in skeletal muscles^3^. 

Thus, functional capacity is limited not only by cardiac factors but also by the interaction between the cardiopulmonary and locomotor complexes^4^. Abnormal ventilatory responses, which are common in patients with HF^4^, combine with peripheral muscle dysfunction to reduce the patients' ability to perform daily life tasks and impair their quality of life^4^^,^ 5, directly affecting their ability to perform physical exercise. Although studies have shown direct benefits from regular physical training in patients with HF by promoting a gradual improvement in tolerance to effort^6^, increased maximal oxygen consumption (VO_2_)^7^, and increased oxidative capacity of the skeletal muscles^8^, the blood flow to the respiratory muscles tends to rise significantly during exercise training because of the redistribution of blood flow from the locomotor muscles^9^. Consequently, reduced oxygenation of the peripheral muscles and elevated lactate concentration occur, increasing muscle fatigue^9^. 

Accordingly, noninvasive ventilation (NIV) may reduce the respiratory work required and increase the physical performance of such patients^10^ by increasing the oxygenation in peripheral muscle microcirculation and improving local blood flow^11^, in addition to possibly improving oxygenation by increasing transpulmonary pressure, which facilitates alveolar ventilation^12^. Similarly, NIV may lead to increased intrathoracic pressure, lowers the left ventricular transmural pressure, reducing preload and afterload, helping to improve cardiac function and providing relief from symptoms of HF^13^. 

Continuous positive airway pressure (CPAP and bi-level positive airway pressure (commercially known as BiPAP*)*, characterized by promoting constant pressure during inspiration and expiration^14^^,^ 15 or positive inspiratory and positive pressure^16^^,^ 17, respectively, are known as NIV therapies. 

This study aimed to conduct a systematic review with meta-analysis of controlled, randomized clinical trials that evaluated the effects of NIV on exercise tolerance in patients with HF because there is no consensus data on the outcome of these methods in exercise intolerance. 

## Method

The systematic review was performed according to the recommendations of the Preferred Reporting Items for Systematic Reviews and Meta-Analyses (PRISMA)^18^. 

### Inclusion criteria

This review included controlled and randomized clinical trials conducted in patients with HF, regardless of functional class and etiology. Inclusion criteria: subjects aged over 18 years of age, with no associated chronic obstructive pulmonary disease or sleep disorders, using NIV associated or not associated with other therapies, including physical exercise. The included studies had to examine exercise tolerance as a primary outcome, analyzing at least one of the following variables: distance travelled during the 6-minute walk test (6MWT), shuttle test or peak oxygen consumption (VO_2_ peak), maximal oxygen consumption (VO_2_ max), exercise time, or dyspnea. There was no language restriction for the search, and all included studies were translated when necessary and possible. 

### Exclusion criteria

Exclusion criteria included studies with unclear or poorly described randomization processes; unclear, inadequate or poorly described interventions; published only in abstracts. 

### Search strategies

The search for scientific articles was conducted by independent researchers in the online databases MEDLINE (via OvidSP), PEDro and SciELO from the start of the databases until June 2013 and was structured in the PICO format, an acronym that stands for patient, intervention, control, outcome*,* based on the PRISMAsearch strategy recommendations^18^. The search was based on words from the Medical Subject Heading Terms (MeSH) dictionary, descriptors, and Boolean operators. The first search was conducted in the MEDLINE database (via OvidSP) as follows: [(heart failure/ or congestive heart failure.tw) and (CPAP ventilation.tw or continuous positive airway pressure/ or bilevel positive airway pressure.tw or positive pressure respiration/ or no invasive ventilation/ or CPAP) and (exercise/ or aerobic exercise/ or rehabilitation/) and (exercise tolerance/)]. The searches in subsequent databases were adapted depending on the database used, and the details of these adapted searches may be requested from the authors. A manual search was conducted of the references of the articles included to complement the search. 

### Selection of studies and methodological quality

Two independent assessors analyzed the search results to find potentially eligible studies. The studies were initially selected based on the title; then, the abstracts were analyzed, and only potentially eligible studies were selected. Based on the abstracts, the full papers were acquired for further selected study. In the case of disagreement between the assessors, a third assessor made the decision on the eligibility of the study in question. 

The methodological quality of the selected studies was evaluated according to the PEDro scale^19^. The PEDro scale comprises 11 items designed to evaluate the methodological quality - internal validity and statistical information - of randomized clinical trials. Each item satisfied, except for the first, scores one point for the overall final classification (0 to 10 points). Two experienced assessors analyzed each study independently and rated it when such a rating was not available in the PEDro database. Discrepancies between assessors were resolved by a third assessor.

### Data collection and analysis

Data collection and analysis was conducted by two authors independently, and the results were expressed as the mean and standard deviation and compared by a third author to assess the accuracy of the data collected. A meta-analysis was conducted regarding similarity among studies in the intervention used, patient characteristics, and variables analyzed. A single meta-analysis was possible, and the mean and standard deviation data of the studies included were extracted and converted into mean differences (treatment effect) and 95% confidence intervals (95% CI). The statistical homogeneity among studies was assessed using the I^2^ test value to determine whether the positive pressure used in the CPAP group affected the response to 6MWT compared to the control/placebo. The only possible meta-analysis was performed using a fixed effects model because significant heterogeneity was not expected. Such meta-analysis was conducted using the Review Manager 5.2 software. The studies that precluded conducting this type of analysis were individually evaluated by analyzing the methodological quality and the data reported in each study. 

## Results

**Figure 1:**
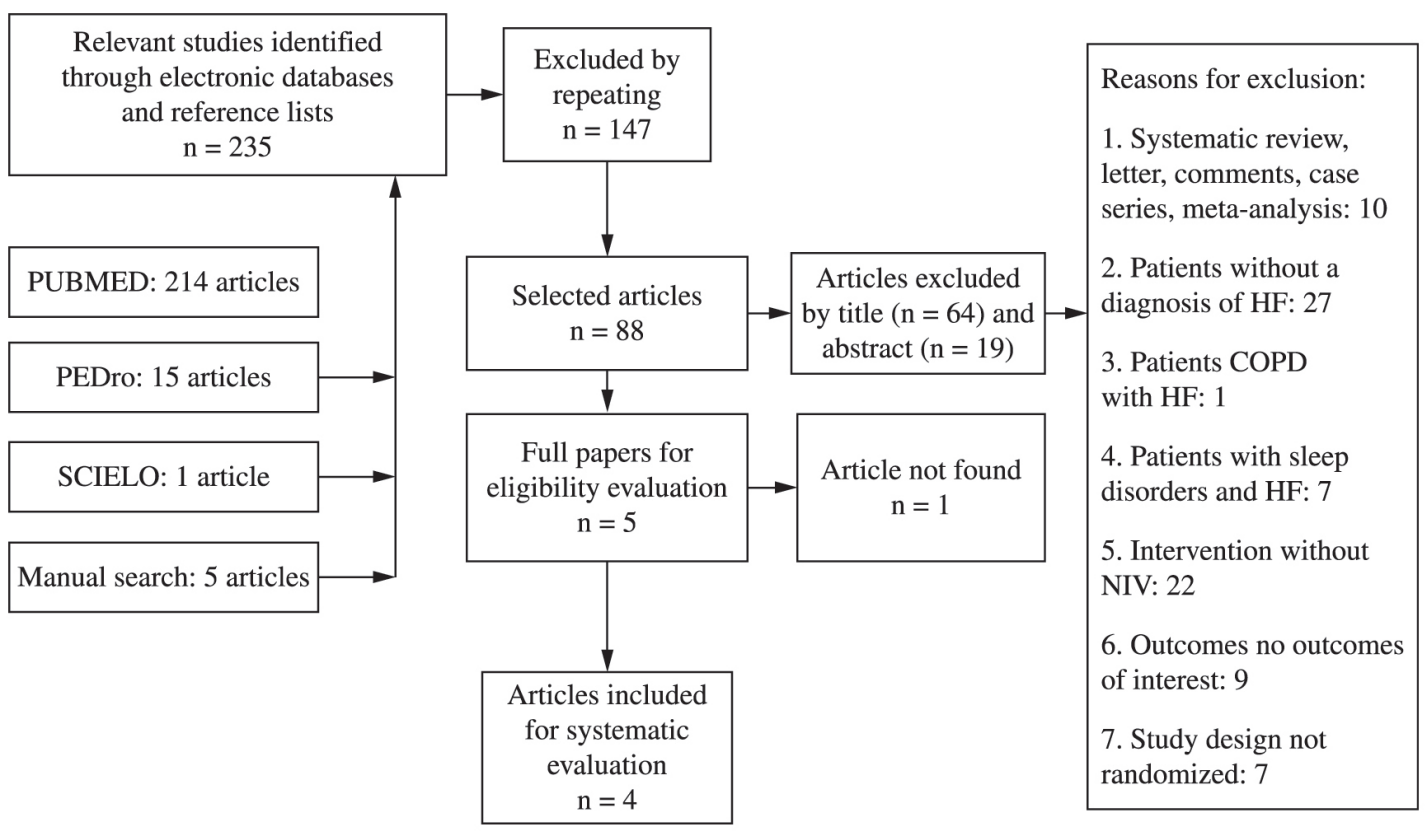
Flow chart of the search process.

Atotal of 235 articles were identified in the search (Figure 1), of which 88 were selected for evaluation based on the title, and their respective abstracts were reviewed. Five articles were eligible for a full review based on the abstracts, and a total of four articles met all inclusion criteria (Table 1). 

**Table 1:** Description of the studies included in this review (p values for comparison between groups).

AUTHOR and YEAR	SUBJECTS	ASSESSMENT / INTERVENTION TIME	PROTOCOL	MEASUREMENTS / RESULTS			
				6MWT	VO_2peak/max_	Testing time	Dyspnea
O’Donnell et al.^20^ 1999^¥^	Total=12 Men=11 Women=1	During/after CPET	During CPET: I1 (n=12) CPAP 4.8 cmH_2_O I2 (n=12) PS 4.8 cmH_2_O I3 (n=12) control 1 cmH_2_O	NA	NA	I1: 8.7±1.1 min I2: 10.1±1.5 min I3: 7.2±1.0 min I1 vs. I2 (p>0.05) I1 vs. I3 (p:0.08) I2 vs. I3 (p<0.01)	I1: 5.1±0.5 I2: 5.5±0.5 I3: 5.2±0.5 I1 vs. I2 (p>0.05) I1 vs. I3 (p>0.05) I2 vs. I3 (p>0.05)
Wittmer et al.^21^ 2006	Total=22 Men=12 Women=10	before, 4th, 9th, and 14th intervention days	2 w; 1 x/day G1 (n=12) CPAP 8 cmH_2_O 30 minutes+ breathing exercises G2 (n=10) breathing exercises	G1: 28% (18-38%)^a^ G2:0% (p<0.01)	NA	NA	NA
Chermont et al.^4^ 2009^¥^	Total=12 Men=8 Women=4	before/after 6MWT	I1(n=12) CPAP 4 to 6 cmH_2_O 30 minutes before 6MWT I2 (n=12)= placebo 0 to 1 cm of H_2_O before 6MWT	I1: 507±3 m I2: 446±36 m (p<0.001)	NA	NA	NA
Lima et al.^5^ 2011	Total=12 Men=8 Women=4	before/after 6MWT	G1 (n=6) CPAP 10 cmH_2_O 30 min before 6MWT G2 (n=6) 6MWT without previous CPAP	G1: 534±89.9 G2: 420.6±73.8 (p:0.03)	NA	NA	G1: 11±0.8 G2: 13±1.1 (p:0.009)

Distance walk; 6MWT: six-minute walk test

**VO2peak/max:** : peak oxygen consumption or maximal oxygen consumption

CPET : cardiopulmonary exercise testing

NA : not available

CPAP : Continuous Positive Airway Pressure

PS : pressure support

w .: weeks

¥ Randomized controlled (cross-over)

G1 : group 1

G2 : group 2

I1 : intervention 1

I2 : intervention 2

I3 : intervention 3

a standard error

The sample size of the studies included ranged from 12 to 22 subjects and involved 58 subjects in total. Comparing the treatments showed that the study by O'Donnell et al.^20 ^used different forms of NIV and a control group; Lima et al.^5 ^and Chermont et al.^4 ^used a control group and placebo, respectively; whilst Wittmer et al.^21^ applied another form of treatment for comparison with NIV. 

The evaluation of the methodological quality of the studies included is described below (Table 2). 

**Table 2:** Methodological classification according to the PEDro scale.

Criteria	O’Donnell et al.^20^, 1999	Wittmer et al.^21^, 2006	Chermont et al.^4^, 2009	Lima et al.^5^, 2011
Eligibility criteria	No	Yes	No	Yes
Random selection	Yes	Yes	Yes	Yes
Secret allocation	No	No	No	Yes
Homogeneity before treatment	No	Yes	No	Yes
Blind subjects	No	No	No	No
Blind therapists	No	No	No	No
Blind appraisers	No	Yes	Yes	No
Appropriate monitoring	Yes	Yes	Yes	Yes
Intention to treat	No	No	No	No
Comparison between groups	Yes	Yes	Yes	Yes
Punctual and variability measurements	Yes	Yes	Yes	Yes
TOTAL	4/10	6/10	5/10	6/10

The eligibility criteria item is not included in the final score.

### Methodological quality

Two of the four studies selected^4^^,^ 5 ^,^ 20 ^,^ 21 were indexed in the PEDro Scale^4^^,^ 21 and had a score available in the database. Two studies^5^^,^ 20 were scored after reading the papers fully and consensus among the three assessors was achieved. The assessors defined the scores corresponding to values from 7 to 10 as high-quality studies, 5 and 6 as of intermediate quality, and from zero to 4 as of low quality. 

The scores ranged from 4 to 6 points on a 0-10 scale. All studies lost points on items related to blinding of the patient and therapist, and only two blinded the assessor. The minimum score of 4 points was found in one study^20 ^_, _and the maximum score of 6 points was only found in the study by Lima et al.^5^. 

### 6-minute walk test

Three of the four studies that evaluated patients with HF subjected to NIV, with or without exercise, evaluated the 6MWT, and all showed positive results^4^^,^ 5 ^,^ 21. Wittmer et al.^21^ compared a program of respiratory exercises associated with 30 minutes of CPAP (8 cmH_2_O) *versus* respiratory exercises only for a period of 14 days; Chermont et al.^4^compared 30 minutes of CPAP (4 to 6 cmH_2_O) *versus* placebo prior to 6MWT, and Lima et al.^5 ^showed that using CPAP (10 cmH_2_O) for 30 minutes prior to 6MWT increased the distance traveled compared to the control without using CPAP (Table 1). 

### Maximal/peak oxygen consumption and exercise time

None of the studies evaluated the VO_2_peak/max. The study by O´Donnell et al.^20^ was the only one that evaluated the use of NIV during the exercise test, showing that exercise endurance in the test with CPAP, with inspiratory pressure support, and without any noninvasive ventilatory support (control) in randomized order was similar to the exercise endurance of the same subjects in the test with inspiratory pressure support and CPAP, although a difference occurred when comparing pressure support to the control. 

### Dyspnea

Two studies evaluated dyspnea when performing the functional capacity test^5^^,^ 20. The only one that showed improvement was Lima et al.^5^, wherein patients reported a lower sensation of dyspnea after running, having used CPAP 30 minutes before the 6MWT, compared to patients who performed the test without using CPAP, as assessed by the Borg scale^22^. 

### Effects of NIV with CPAP mode on exercise tolerance in the 6-minute walk test

**Figure 2:**
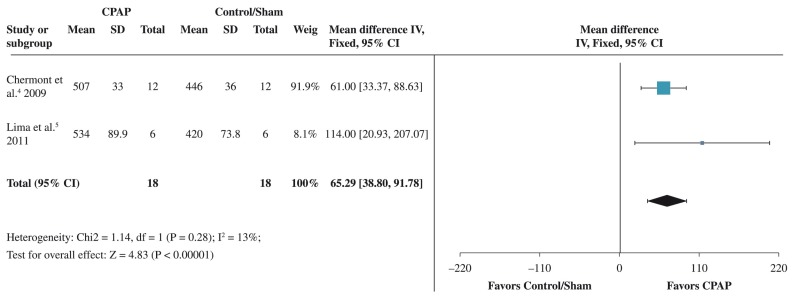
Forest plot of the results of the meta-analysis. Comparison of intervention using positive pressure with CPAP versus a control/ sham group to test the outcome. The values shown are the average effects (difference between means) with a confidence interval of 95%. The average result was calculated using a fixed-effect model.

Although three studies^4^^,^ 5 ^,^ 21 evaluated the 6MWT, the data from only two^4^^,^ 5 could be grouped for analysis. The analysis of heterogeneity resulted in an I^2 ^value of 13% (p=0.28), which showed low heterogeneity between studies. The difference found was 65.29 (from 38.80 to 91.78), with 95% CI. A greater weight was assigned to the study by Chermont et al.^4^, corresponding to 91.9%. That result favored the use of CPAP in improving the distance travelled in the 6MWT (Figure 2). 

## Discussion

Patients with HF usually exhibit dyspnea and fatigue during exercise and/or daily life activities as their main clinical symptoms^2^^,^ 4. The progression of these symptoms, which link the neurohormonal changes to declining ventricular function during the process of myocardial and vascular remodeling and also to increasing lung congestion, promotes a decreased level of physical activity that leads to lack of physical fitness^23^. Reduced physical fitness contributes to further worsening of the symptoms, limitations of daily life activities, and exercise intolerance, progressively reducing functional capacity, which leads to a frequent clinical condition associated with high costs, disability, and high mortality rates^1^. 

Functional capacity has been widely evaluated in research studies and under clinical conditions in such patients^24^, and the 6MWT is a simple, low-cost method of evaluating that parameter. The 6MWT test is able to reproduce daily life activities^24^, evaluate exercise tolerance^4^^,^ 25, assess the degree of functional limitation, and enable prognostic stratification^25^^,^ 26. 

This systematic review with meta-analysis showed that the effect of a single session of NIV with CPAP mode prior to exercise promoted an increase in functional capacity as expressed by the increased distance traveled in the 6MWT by patients with HF^4^^,^ 5. 

The use of NIV may be able to promote the decrease in pre- and post-load on the ventricles, increasing the ejection fraction of the left ventricle (LV) and reducing myocardial oxygen consumption associated with the production of carbon dioxide^13^^,^ 27. The improvement in cardiovascular function may be confirmed as a result of CPAP-induced increase in intrathoracic pressure, wherein positive pressure decreases the large variations in pleural pressure. This decrease reduces the transmural pressure of the left ventricle (LV), thus improving the contractile performance of the heart^13^. NIV with CPAP also decreases the peripheral vascular resistance, which contributes to reducing the LV post-load. Altogether, these effects may decrease systolic blood pressure, although the overall perfusion of peripheral tissue is high, which shows greater cardiocirculatory efficiency^13^. However, the ventilatory and hemodynamic effects of the application of a single session of NIV before effort to increase exercise tolerance are still not fully known. 

Chermont et al.^4 ^showed that NIV with CPAP decreased the resting heart rate (HR) and blood pressure (BP) and increased the distance travelled during the 6MWT compared to the placebo group which is similar to the results found by Acosta et al.^28^. Furthermore, the authors showed an increase in peak HR during the 6MWT, showing an increase in the chronotropic reserve, suggesting that this effect was involved in the mechanism responsible for the increased exercise capacity, among the wide variety of responses. Previously, Reis et al.^29^ showed that the acute effects of using CPAP, both at 5 cmH_2_O and at 10 cmH_2_O, promoted improvement in the autonomic modulation of the HR of patients with chronic HF^29^. However, the authors failed to assess whether the effect continued after withdrawing the NIV support. 

Although Lima et al.^5^showed no significant effects on hemodynamic parameters (HR and BP) from the prior use of positive pressure compared to the control group at the end of the 6^th^ minute, in the evaluation of the double product at the end of the exercise, lower values of that physiological property, improved SpO_2_, and reduced dyspnea were observed in the group of patients who used CPAP prior to the 6MWT. That finding indicates that NIV may be able to improve the performance of the cardiovascular and respiratory system beforehand, so that such patients show lower cardiac work when performing the 6MWT. 

Regarding the use of NIV support for a longer period, Wittmer et al.^21 ^showed that using CPAP associated with respiratory exercises for 14 consecutive days increased pulmonary function and the distance travelled in the 6MWT. Positive pressure increases pulmonary compliance, improves gas exchange, reduces respiratory effort, and reduces airflow limitation by decreasing airway resistance in patients with HF^21^. These results corroborate the results of Yan et al.^30^, showing the ability of NIV to improve the parameters of pulmonary function and, thereby, enabling exercise to be performed more effectively^30^. However, a thorough analysis was hampered by the lack of similar studies that might have reinforced the notion that daily CPAP sessions would be able to maintain their effect for an extended period. 

The only study^20^ that evaluated the use of NIV during an exercise test showed that NIV support reduced discomfort in the lower limbs and increased exercise resistance, with an increase of 2.8 minutes in the use of inspiratory pressure support (PS) during the stress test compared to non-use of ventilatory support. The authors reported that increased exercise time could be explained by the decreased discomfort in the lower limbs, which was assessed by the Borg scale during the use of NIV. Another possible mechanism corroborates the findings of Borghi-Silva et al.^11^, and O'Donnell et al^20^ who claimed that PS could improve peripheral perfusion and muscle oxygenation fraction by improving cardiac output and/or by altering regional vascular distribution. Significant improvement was observed in the time of the ergospirometric test, 2.55 minutes on average, in the study of ten cases of HF conducted by Azevedo et al.^31^. Lower values (1.38 minute) were observed by Steiner et al.^32 ^in the use of nocturnal CPAP (>6 h) after approximately eight months of intervention. 

Functional capacity is usually measured by a cardiopulmonary exercise test that measures exercise tolerance and helps in the diagnosis of HF^33^. None of the studies included in this review evaluated the VO_2_max/peak, most likely because they were short-term studies. Furthermore, no study used NIV associated with an exercise protocol, which could contribute to increasing the VO_2_max/peak, which is improved by regular physical exercise, particularly with training intensity sufficient to generate cardiovascular fitness, especially in the relevant population^1^^,^ 34 ^,^ 35. 

Dyspnea is one of the most important variables analyzed for the diagnosis of cardiorespiratory disease because it manifests itself when patients perform their routine activities^2^ and, in more severe cases, may progress to dyspnea at rest^1^. Two studies included in this review analyzed dyspnea^5^^,^ 20, and only Lima et al.^5 ^showed that using CPAP for 30 minutes (10 cmH_2_O) could decrease post-6MWT dyspnea in comparison to the group that did not undergo prior NIV. In a non-randomized study conducted by Bussoni et al.^36^, 11 patients with HF took the 6MWT before and after using CPAP (30 minutes, 10 cmH_2_O), and the results showed that lower dyspnea occurred at the end of the second test. However, the small number of subjects precluded us from confirming whether the results were reproducible when including a larger number of participants. 

Another issue requiring further study is the ideal positive pressure to promote increased exercise tolerance. Some authors advocate a CPAP value of 10 cmH_2_O^37^ because it promotes alveolar recruitment and effective gas exchange, whilst others advocate smaller values, namely 6 cmH_2_O or the value best tolerated by patients^4^^,^ 38 because a significant improvement in cardiac output is shown with low levels of CPAP. 

Although most studies that evaluate the effect of the final positive pressure use a value of 10 cmH_2_O^5^^,^ 31 ^,^ 36 ^,^ 39 ^,^ 40, regardless of outcome, and although that value is recommended by the Brazilian consensus on mechanical ventilation for patients with acute HF^37^, such recommendations are not quite as well established for stable patients who are or who need to start practicing physical exercise. 

Accordingly, our meta-analysis showed that the CPAP values used by Chermont et al.^4 ^(4-6 cmH_2_O) and Lima et al.^5 ^(10 cmH_2_O) favorably promoted an increase in the distance traveled during the 6MWT. Although the methodological quality of the study by Chermont et al.^4 ^(5/10) was lower than the quality of the study by Lima et al.^5^(6/10), greater weight was assigned to the results of the study by Chermont et al.^4 ^(91.9%). The authors indicated that patients might not adapt to nor require pressures close to 10 cmH_2_O. Thus, our meta-analysis suggested that lower pressures might promote favorable effects regarding exercise tolerance, thus avoiding discomfort that often leads to a lack of adherence to treatment. 

Our findings showed that no published studies have examined the use of NIV with two levels of pressure (only support pressure^20^) in exercise tolerance in patients with stable HF. Such findings are most likely because that ventilatory mode is more recent and is preferentially used in patients with the presence of hypercapnia^41^. 

Thus, the results from this meta-analysis suggest that using NIV in the CPAP mode 30 minutes before the 6MWT may increase tolerance to exercise and that a final positive pressure below 10 cmH_2_O may also have a beneficial effect on the increase in the distance travelled. Therefore, our results may be used as a key complement to the literature for professionals working in cardiac rehabilitation services. 

### Study limitations

The limitations may be described as a result of the small number of studies that could be included in the systematic review because indexed articles on this topic are scarce, which directly affected the meta-analysis. 

Only two studies could be used for a single meta-analysis on the outcome distance travelled in the 6MWT, and despite their low heterogeneity and positive results in the 6MWT, these studies were of limited accuracy due to the small number of participants. Similarly, the methodological quality of studies in the PEDro Scale was intermediate to low, thus limiting a "safe" conclusion. Therefore, any effect estimates were very uncertain, and further research studies may impact such estimates, which suggests that new studies should be conducted with a larger number of patients. 

## Conclusion

This review with meta-analysis suggests that the use of NIV before the 6MWT in patients with HF may promote an increase in the distance travelled. However, the limited number of studies preclude a more thorough analysis of the effects of NIV on exercise tolerance in these patients. 
